# Optimizing noisy galvanic vestibular stimulation (nGVS) for postural control: methodological considerations when individualizing the signal for people with bilateral vestibulopathy

**DOI:** 10.3389/fneur.2025.1609123

**Published:** 2025-06-20

**Authors:** Ruth McLaren, Paul F. Smith, Rachael L. Taylor, Denise Taylor

**Affiliations:** ^1^School of Clinical Science, Auckland University of Technology, Auckland, New Zealand; ^2^Eisdell Moore Centre for Hearing and Balance Research, University of Auckland, Auckland, New Zealand; ^3^Department of Pharmacology and Toxicology, Faculty of Biomedical and Molecular Sciences, The Brain Health Research Centre, University of Otago, Dunedin, New Zealand; ^4^Department of Physiology, University of Auckland, Auckland, New Zealand; ^5^New Zealand Dizziness and Balance Centre, Auckland, New Zealand

**Keywords:** neuromodulation, galvanic vestibular stimulation, nGVS, vestibular, posture, balance, stochastic resonance, optimization

## Abstract

An established aspect of noisy galvanic vestibular stimulation (nGVS) is tuning the nGVS signal to optimize stability on an individual basis. However, conventional tuning methods are strongly influenced by historical approaches and fail to integrate contemporary research findings. We outline a process used to integrate current physiological and neuroscientific insights into a robust method for personalizing nGVS signals to improve stability. We argue that an optimization protocol for a neuromodulatory nGVS signal designed to facilitate postural control needs to include: (1) A task that is relevant to the population, and which can be modified to give an appropriate level of challenge at an individual level; (2) Elements that can be reliably measured and are responsive to changes in postural control; (3) Well controlled and defined signal parameters; (4) Potential to be translated into the clinical setting. Questioning conventional methods enabled us to develop an alternative nGVS optimization assessment to enhance postural control in people with bilateral vestibulopathy. Refining this optimization assessment represents a crucial step in developing individualized nGVS interventions. The fundamental principles applied to develop our method can be adapted to other neuromodulatory stimuli across different impairments and populations.

## Introduction

Postural control refers to our ability to maintain, achieve or restore a state of balance (1). Adequate postural control is a prerequisite for independent mobility, and if lacking can lead to reduced mobility, loss of confidence, imbalance, falls, injury, and social isolation (2, 3). There are large social and economic costs associated with poor balance (4). On this basis, interventions to restore postural control have been explored extensively (5, 6). Noisy galvanic vestibular stimulation (nGVS) is one treatment option that has been investigated. While nGVS has been found to improve postural control in research trials (7–27) and meta-analyses (28, 29), results have not been consistent. The dynamic and interdependent nature of postural control mechanisms, alongside the diverse signal parameters and varied methods of signal optimization, appear to contribute to the discrepancies noted in research (30).

nGVS is a stochastic noisy neuromodulatory stimulus, typically delivered as zero mean and Gaussian via electrodes placed bilaterally over the mastoid process (30). It has been used to facilitate postural control in people with impaired balance stemming from aging or neurological deficits (7, 25, 26, 31, 32). Optimizing the noisy galvanic vestibular stimulation (nGVS) signal to give optimum postural control has been identified as an important component of this neuromodulatory intervention, as the most effective parameters can vary between individuals (30, 31, 33–37). However, despite evidence for the efficacy of nGVS to improve stability, and for the importance of optimizing the signal (28, 30), there has been little research investigating the processes involved in optimizing nGVS to improve postural control.

Central to optimization is the premise that everyone's physiology is unique, thus nGVS parameters must be personalized to achieve the best response. The dominant theory underpinning nGVS is stochastic resonance; the theory that in non-linear sensory systems—i.e., systems characterized by a discrete threshold for sensory transmission—a noisy signal can enhance the detection of weak afferent inputs (38, 39). However, research findings are conflicting, with some studies supporting this theory (25, 31, 40, 41) while others report a response to nGVS that does not follow a stochastic pattern (31, 42, 43). A neuroplastic mechanism may operate alongside the stochastic process. This could help explain postural response patterns that do not follow a stochastic curve, cortical changes observed during and after nGVS, and the sustained effects following stimulation (32, 44, 45).

Conventional optimization methods are strongly influenced by historical approaches. As nGVS research advances, it is important to assess prior assumptions in light of emerging knowledge. In this paper we explore the influence of the task, task challenge, parameter choice, population, responsiveness and choice of outcome measures, on the optimization assessment. We propose an approach to help researchers identify factors that will bolster the optimization process when using nGVS to improve postural control. The overarching principles applied to develop our methodology may apply to other neuromodulatory stimuli that are used to improve postural control.

### What has been tried to date?

To date, three primary methods of tuning the amplitude of the signal to improve postural control have been reported. These methods have varied in time to complete, equipment required and theoretical underpinnings.

Motion perception to a 1 Hz sinusoidal galvanic vestibular stimulation (GVS) waveform has been used to determine a threshold amplitude. A 1 Hz sinusoidal GVS waveform is delivered, and the amplitude at which an individual senses mediolateral motion or, is observed moving on a force plate, is taken as the sensory threshold. A percentage of this threshold amplitude (between 50% and 100%) is then applied to an nGVS waveform (17, 21, 24, 46–50). While a conceptually sound method to investigate the responsiveness of the vestibular system, there is no evidence for a commensurate relationship between the motion perception threshold to a sinusoidal signal and a postural response to a noisy signal (49). Although this method has been used historically, it has fallen out of favor in recent years. It also requires equipment that can provide both a sinusoidal and a noisy signal at a variety of amplitudes; this type of equipment is not readily available at present.

The cutaneous nGVS threshold has been used as a quick, simple method of optimization, appealing for research and clinical practice (15, 16, 27, 51). The cutaneous threshold is determined by finding the point at which nGVS elicits cutaneous sensation under the electrodes (15, 16, 27, 51). Stimulation is then delivered at around 80% of this cutaneous threshold. Hesitancy exists around this method as the relationship between cutaneous sensation and vestibular function is unclear (30). Sensation from the skin over the mastoid process, travels through the posterior branch of the auricular nerve, through the dorsal root ganglion to the C2/3 spinal root, then ascends to synapse in the medulla, before transmission to the sensory cortex. In contrast the vestibular system sends signals from the vestibular apparatus via the vestibulocochlear nerve, to the vestibular nuclei in the brainstem prior to making diffuse connections within the brain, including the cerebellum and hippocampus (52). In addition, the threshold for cutaneous sensation is influenced by a complex interplay of physiological, environmental and temporal factors. Stress levels, circadian rhythm and airborne allergens, are among some of the factors that can influence sensory perception, mitigating the reliability of this method (53–55).

The most direct method for optimizing nGVS stimulation is to present nGVS at different amplitudes to identify the point at which postural stability is maximally enhanced (7, 8, 20, 22, 23, 42, 49, 50, 56). Although time intensive, this method has been the most frequently employed (30), most closely aligns with the principle of stochastic resonance, and serves as the gold standard in comparative studies (8, 19, 49, 50). Wuehr et al. (26) applied various amplitudes to optimize nGVS in people with bilateral vestibulopathy (BVP). Their findings revealed that most participants exhibited stochastic response patterns. This supports the theoretical foundation for using postural control measurements across different amplitudes to identify the optimum stimulation amplitude. While three different optimization methods appear in the nGVS literature, directly testing postural stability across a range of amplitudes remains the most conceptually sound approach. Although this method requires more time, this disadvantage is offset by the greater certainty it provides in determining the optimal stimulation parameters.

### What factors should we consider in task selection?

Postural stability requires appropriate motor output in response to multisensory integration of vestibular, visual, and proprioceptive afferent information. Historically, the majority of nGVS studies have focussed on optimizing the stimulation signal based on changes observed during standing (30). However, loss of standing postural control is seldom mentioned in the clinical BVP literature (2). The Bilateral Vestibulopathy Questionnaire does not include standing balance, and the Oscillopsia Functional Impact Scale has only one question referencing standing, out of a total of 43 (57, 58). In contrast, gait instability is widely recognized as a defining feature of BVP (59). In the Bilateral Vestibulopathy Questionnaire five of the 20 questions relate to gait (57) and in the Oscillopsia Functional Impact Scale 12 of 43 questions refer to gait (58). Recent preliminary research indicates that responses to nGVS may be task specific. Peto et al. (60) found that signals optimized in a standing position had no effect during gait in individuals with Parkinson's disease. Further research is required to determine whether this task specific response to nGVS is generalized to other populations. We propose that in studies to improve postural control, choosing an optimization task that is relevant to the individual and their deficit is preferable.

Typically, nGVS optimization in standing has utilized the velocity of the center of pressure during postural sway, sometimes combined with sway area and root mean square (RMS) displacement (30). In standing the body is commonly modeled as a single-segment inverted pendulum pivoting around the ankle (61). Quiet stance is characterized by body sway produced by gravity acting on the center of mass and by intrinsic forces. Equilibrium is maintained primarily by steady state control, where the musculoskeletal system makes small postural adjustments to maintain stability. Postural sway gives us insight into the sensory-motor integrity of the nervous system in a situation where the primary destabilizing forces are gravity and small internal forces (62). The argument for using postural sway as an optimization task is that limiting sway is advantageous to a point. Reducing mediolateral sway appears to be particularly beneficial to maintain balance (1, 63). While using standing sway as an optimization task has merit, it is reliant on the assumption that less sway is better. In reality, there is a “Goldilocks zone” for sway. Too much sway can indicate insufficient sensory information is available for the sensitive and fine control required to maintain quiet standing. Conversely, too little sway can indicate the individual is using excessive rigidity to maintain postural control and lacks the flexibility characteristic of a healthy biological system (64–66). Thus, a reduction in sway may not always indicate improved postural control. In addition, while imbalance and oscillopsia are the primary deficits reported in BVP (2), patients seldom report that these deficits limit their function in quiet standing (57, 58). Consequently, standing may not be a task that adequately represents situations people with BVP find challenging.

An alternative to standing is the use of gait as the optimization task. A strong argument in favor of this is that the primary deficit reported by people with BVP is imbalance during gait (2, 3, 67). This reinforces the role of the vestibular system in more dynamic activity involving greater head movement and postural challenge. Gait requires the coordination of numerous muscles and joints to progress forward, orientate body segments and adjust to environmental demands (68). Vestibular information regarding the acceleration and translation of the head is integrated with afferent inputs from the visual, and somatosensory systems (69). During gait the center of mass sits outside the base of support for 60–80% of the gait cycle, making the task inherently unstable (53, 70). We maintain that, for people with BVP, assessing spatiotemporal gait parameters is a more relevant and meaningful task on which to base neuromodulatory signal optimization.

### What aspects of task challenge do we need to consider?

Task challenge appears to affect the ability of nGVS to influence postural control (71). When an optimization task is too easy, the vestibular system already has capacity to meet task demands. For example, nGVS has no effect on postural control when healthy individuals walk across a well-lit room (22). The healthy vestibular system has sufficient capacity to easily meet the demands of the task, and nGVS facilitation has no effect on motor output. In contrast, when people with BVP receive nGVS facilitation walking in a well-lit room, their gait stability improves (22). The gait task challenges the capacity of people with BVP; thus, facilitation of the vestibular afferent signal improves gait stability.

At the other extreme, a highly challenging task can exceed the capabilities of the postural control system to the point where despite an nGVS boost to the vestibular system, the system will still fail. For example, nGVS failed to improve postural control when people with BVP stood on foam with their eyes closed (21). An extremely challenging task such as this may be so far beyond the individual's capacity there is not a measurable response to an enhanced vestibular signal. Consequently, a task that is too easy or too hard can obscure the potential benefit of neuromodulation.

Individual capacity influences task challenge (71). Overground walking is sufficiently challenging to see the effects of nGVS in most people with BVP, making it a suitable task (8, 22). However, higher functioning participants may require a more challenging option (69, 72, 73). Thus, it is worthwhile considering how the task may be modified to provide an ideal level of challenge. A hallmark of BVP is imbalance when visual input is reduced, or somatosensory information becomes less reliable (59). Therefore, we can consider how these components can be manipulated during the optimization task. For example, walking with eyes open or closed will alter the visual condition, and the choice of surface may influence the somatosensory feedback. Similarly, modifying gait speed may offer an alternative approach to influence task challenge. Slow gait speeds are associated with increased gait variability in people with BVP (69, 72), as well as greater responsiveness to nGVS (15).

### What aspect of gait should we measure, and how does this relate to the output we are modulating?

Historically, gait motor control theory has been dominated by the role of central pattern generators; neuronal circuits at a spinal level that can produce rhythmical motor patterns, such as those used in gait (74). While the central pattern generator has a crucial role to play, this focus has led to less emphasis on the influence of afferent sensory feedback, and cortical control which enables variability and flexibility in the gait cycle (75). This variability and flexibility, particularly the ability to make precise adjustments to foot placement and step timing are prerequisites for walking in the real world (76, 77).

The spatiotemporal parameters of gait have been divided into domains that represent characteristics of gait: namely, pace, rhythm, variability, asymmetry and stability. People with BVP demonstrate marked changes in the domains of pace, variability and stability (69, 73, 75, 78–80). The gait patterns observed in individuals with BVP typically involve spatial and temporal adaptations that build more stability into the gait pattern. People with BVP tend to have a slower preferred gait speed, with shorter quicker steps- this reduces the duration of time where the center of mass lies outside of the base of support (8, 69, 80–82). Steps tend to be wider, with a larger proportion of the gait cycle spent in double support. This improves lateral stability and increases the opportunity to use motor strategies to control the center of mass (69, 79, 81, 82). Gait is also thought to become more variable in the absence of vestibular information. Studies have found people with BVP demonstrate more variability in foot placement, with higher standard deviations and coefficients of variation of stride time, step length and step width (69, 72, 78, 81). Changes to the spatiotemporal parameters of gait are hypothesized to result from the lack of vestibular afferent information affecting feedforward mechanisms of motor control and the fine tuning of foot placement (75, 76, 83).

There is little work investigating optimization of nGVS using gait. Mulavara et al. (49) used a cost function derived from 7 gait stability measures during perturbed walking at different nGVS signal amplitudes. This method was complex, requiring extensive equipment and calculations and has not been repeated in the literature. Iwasaki et al. (8) used the nGVS amplitude that resulted in the greatest increase in gait speed. This decision was based on the premise that the vestibular system plays a role in maintaining a consistent pace (75). In contrast to the complex approach adopted by Mulavara et al. (49), the Iwasaki et al. (8) approach may *oversimplify* the vestibular impact on gait. Although people with BVP typically have a slower preferred gait speed (72), they may periodically maintain or even increase walking velocity to reduce their reliance on afferent sensory information (73, 82).

To find a middle ground between existing approaches, we propose a theoretically informed methodology for parameter optimization based on assimilation of the gait and BVP literature. Lord et al. (84) divided gait into five key domains: pace, rhythm, variability, asymmetry, and stability (Table 1). Using the BVP literature, we identified key spatiotemporal features of gait affected when vestibular afferent information is lacking and categorized them by domain (Table 1) (75). We hypothesized that if these gait parameters were influenced by the absence of vestibular signals, then restoration of these afferent signals would change the parameters in the direction of improved stability. By interpreting the BVP literature through the lens of gait theory, we were able to combine theoretical understanding with practical application to develop a robust approach to optimization assessment (Table 2).

**Table 1:** Changes in gait pattern characteristic of BVP.

**Domain**	**Gait parameter**	**Change**	**Lang et al. (18)**	**Schniepp et al. (72)**	**Schniepp et al. (69)**	**Iwasaki et al. (8)**	**McCrum et al. (81)**	**Herssens et al. (82)**	**Grouvel et al. (78)**	**Grouvel et al. (79)**
**Pace**										
	Cadence	↑	X					X		
	**Gait speed**	↓			X	X				X
	Step length	↓			X		X			X
	Stride length	↓				X	X			
**Rhythm**										
	Step time	↓						X		
	Stride time	↓				X				
	Stride time CV	↑		X	X					
**Variability**										
	Step length CV	↑			X		X			
	Gait SD	↑							X	
**Stability**										
	**Step width**	↑			X		X			X
	**Step width CV**	↑					X	X		
	**Double support time**	↑					X			X
	Trunk sway	↑	X							

CV, Coefficient of variation; SD, Standard deviation.

**Table 2:** Application of the theoretical optimization framework to an nGVS optimization protocol for people with bilateral vestibulopathy.

**Question defined in framework**	**Options considered**	**Decision made**
What methods of optimization have been tried to date?	Cutaneous threshold Motion perception threshold	While three different methods of optimization have been used in the nGVS literature, the most direct method, of testing postural stability over a range of amplitudes appears to align with the underlying principle of stochastic resonance. The disadvantage of the amount of time involved in this method is mitigated by greater certainty of this approach.
	Test array of values and measure outcome	
What postural control tasks should we consider?	Standing	We suggest that assessing spatiotemporal gait parameters is a more relevant and meaningful task than standing sway for optimizing assessment in people with BVP. Gait is able to capture the functional challenges faced by these individuals in real-world scenarios.
	Walking	
What aspects of task challenge may influence optimization?	Walking eyes open	Overground walking is sufficiently challenging to see the effects of nGVS in people with BVP, making it a suitable task (11, 25). However, higher functioning participants may require a more challenging option (61, 64, 65). A defining characteristic of BVP is imbalance when visual input is reduced (52), therefore, where feasible, it is important to test gait with both eyes open and eyes closed.
	Walking eyes closed	
What aspects of gait should we measure, and how will they respond to neuromodulation?	Pace	Dividing the spatiotemporal parameters of gait that demonstrated change in BVP into their associated domains of pace, rhythm, variability, and stability (Table 1) (75) we identified gait parameters demonstrating a change indicating reduced stability in two or more studies. This generated four variables in the pace domain, one in the variability and three in the stability domains. We considered four pace variables would weight this domain too strongly. Additionally, the gait parameters in the pace domain interact, as gait speed is the sum of cadence and step length. On this basis we decided to use gait speed as a single parameter to represent the pace domain (Table 1), along with stride length CV in the variability domain, and step width SD, step width and double support time in the stability domain.
	Rhythm	
	Variability	
	Stability	
What aspects of measurement do we need to consider?	Data variability	Variability is a characteristic of the BVP population. Balancing reliability against the burden of assessment we require 30 steps as the minimum for reliable variance step data when designing our methodology. Furthermore, the measurement protocol directly influences the minimal detectable change (MDC) and the minimal clinically important difference (MCID). These factors (where available) should be considered when defining the criteria for improved gait stability.
	Reliability of measures	
	Sensitivity to change	
What stimulation parameters should we vary during optimization, and what parameters should we control?	Waveform	We incorporate adjustable parameters informed by evidence-based ranges from our prior work (30). Frequency bands were selected from those demonstrating efficacy at improving stability in people with BVP (0.01–10 and 0.01–30Hz). Amplitude was systematically varied across a range known to elicit improved postural control in vestibular populations (0.1–0.7mA). To isolate the impact of these variables fixed parameters included electrode, location, surface area, skin preparation and interface, the noise distribution and the duration of stimulus. Our optimization protocol minimizes cumulative exposure with total stimulation duration constrained below typical therapeutic doses. nGVS may have a sustained effect after stimulation (79). To mitigate potential carryover effects participants were given a 2-min seated rest between each trial.
	Amplitude	
	Frequency	
	Duration	
	Washout period	
Does this methodology have the potential to translate to a clinical setting?	Duration Equipment Area Processing	If gait is demonstrated to be a reliable and robust method of optimizing an nGVS signal, this method of optimization could be used to inform future trials investigating more portable, time-efficient and space saving technologies, such as accelerometry based, or markerless gait analysis.

The key features of this case study are: (1) Optimization signal: nGVS. (2) Target behavior: Improved postural control. (3) Underpinning theory: Stochastic resonance and neural plasticity. (4) Clinical population: People with bilateral vestibulopathy.

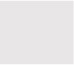
Methodologically sound/important in the context of the theory of stochastic resonance.

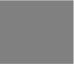
Methodologically sound/important in the context of stochastic resonance and also important for people with BVP.

### Measurement considerations during testing and analysis?

Neuromodulatory optimization studies evaluate within session changes, which are generally modest. Therefore, it is crucial to consider both reliability of the testing methods and how population characteristics might influence the data. In people with vestibular disorders within session test- retest reliability of preferred gait speed is excellent using both manual (ICC = 0.88) and instrumented (ICC = 0.94) recording methods (85, 86). However, the measurement of other gait domains can be challenging as we manage both internal and external sources of variability. Internal sources represent the normal variability of gait as well as variability that represents deficit due to pathological mechanisms (87). Internal variability provides important insights about the stability of gait and the neurological and biomechanical control of posture. It is generally accepted that people with BVP typically exhibit highly variable step time, step and stride length and step width (69, 72, 73, 78, 82).

External variability is the variability that occurs due to measurement error. This variability needs to be minimized to ensure that the variability that is seen in the data primarily represents the internal variability. For example, many studies investigating gait in people with BVP have not reported the number of steps analyzed, and those that do, report using 4–14 steps in their analysis, increasing the risk of external variability in the results (72, 82). It is critical that our methodology is robust and minimizes the risk of measurement error. For example, when measuring variance, research in older adults suggests that between 30 and 220 steps are required to achieve reliable step variance data (88, 89). Of the 5 studies investigating overground walking in people with BVP (8, 69, 72, 78, 79), only one study captured gait over a distance >12 m (69). Therefore, it is unlikely that the volume of steps in these data reached the number required to establish reliable variance measures.

A further cause of external variability can be instrumentation error. A high number of outliers have been reported in the spatiotemporal gait data of people with BVP (69, 72, 73, 78, 79). This can be challenging to manage as outliers relating to the use of foot placement to control the center of mass and maintain postural stability should be retained (internal variability). Conversely, outlier data relating to instrumentation error and not representative of spatiotemporal gait parameters must be removed to maintain data integrity.

### What nGVS parameters should we use, and how may these affect the method?

Guidelines for the stimulation parameters that influence the signal delivered have been covered extensively in our previous paper (30). While nGVS holds promise as a means of improving gait and balance in people with vestibular disorders (28), its transition into clinical practice is hindered by the vast array of different parameters that have been used, and a lack of consensus on optimizing them effectively (30). Parameters such as the electrode surface area, electrode/skin interface, frequency band, amplitude and noise distribution have potential to influence the efficacy of the signal and must be prescribed thoughtfully and reported fastidiously so we can further our knowledge in this area (30). Prior research informs us of parameters and parameter ranges that are likely to be effective, and that require further investigation. Other parameter variables should remain consistent, so the effect of the experimental parameters can be realized.

Additionally, the dose of stimulation over the course of the protocol must remain within safe limits. That is, the total duration of stimulation participants received in the optimization session should remain less than they would receive in a treatment session.

Residual effects and the need for a washout period between stimuli also become important when we are looking for subtle dose-related changes (31). nGVS may have a sustained effect after stimulation (32). The duration of washout between optimization trials has been poorly reported in the nGVS literature with most papers failing to report on the washout period (7, 20, 22, 23, 31, 32, 49, 56, 90). Those that report this metric (8, 19, 22, 42, 48, 50, 91) have a break ranging between 20 s (19, 50) and 3 min (7, 19) between stimulation trials. It has not been ascertained whether these durations are sufficient to eliminate the effects of nGVS. Some studies have found no residual effects (33), while others have noted effects for up to 4 h (7, 14). There is also the potential for cumulative stimulation effects that have not been explored to date. The washout period for stimulation effects warrants further investigation as it has the potential to influence both optimization processes, and the duration of residual facilitatory effects post-nGVS treatment.

### Does this methodology have the potential to translate to optimization in a clinical setting?

While to date, nGVS optimisation has been conducted in research settings, the future of this technology is its adoption in the clinical space. Laboratory- based optimization relies on laboratory equipment such as force plates, motion capture systems and treadmills, which are typically not available in clinical settings due to their high costs, space requirements, and the technical expertise and time required for data acquisition, processing and analysis (30, 92). However, the strength of laboratory-based optimization protocols lies in their ability to generate rich data sets, providing a robust foundation for the development of simpler methods to assess stability in future clinical applications. Emerging technologies, such as body worn sensors, accelerometery and markerless gait analysis systems, are capable of capturing metrics that reliably measure postural stability and show potential as efficient and robust tools for movement analysis within clinical settings (93–95). As nGVS technology approaches clinical feasibility, it is increasingly important to consider how the principles established and validated in the laboratory can be effectively translated into more portable, time efficient and space saving technologies.

## Conclusion

As the importance of individualizing neuromodulation parameters becomes established, it is timely to critically examine current optimization practice, with a view to improve methodological rigor. By critically examining existing methods and incorporating our knowledge of neuroscience and motor control, we have developed a methodology that prioritizes task relevance, appropriate levels of challenge and population specific considerations. Our approach emphasizes the importance of selecting meaningful outcome measures, ensuring data quality and considering the potential for clinical translation from the conceptual stages. The seven key questions proposed in this paper (Table 2), provide a framework for researchers to design rigorous optimization protocols for neuromodulatory stimuli and a foundation for developing individualized interventions.

## Data Availability

The original contributions presented in the study are included in the article/supplementary material, further inquiries can be directed to the corresponding author.
